# Thoracic Outlet Syndrome Managed With Botulinum Toxin Injection Following Multiple Failed Surgeries

**DOI:** 10.7759/cureus.111832

**Published:** 2026-06-30

**Authors:** Brian Mansoury, Ahmed Khawer, Ameer Qadri, Ayah J Syeed, Jason Mizrahi

**Affiliations:** 1 Life Sciences, University of California, Los Angeles, Los Angeles, USA; 2 Physical Medicine and Rehabilitation, Johns Hopkins Medicine, Baltimore, USA; 3 Osteopathic Medicine, Midwestern University Chicago College of Osteopathic Medicine, Downers Grove, USA; 4 Osteopathic Medicine, Ohio University Heritage College of Osteopathic Medicine, Athens, USA; 5 Osteopathic Medicine, Touro College of Osteopathic Medicine, Harlem, USA

**Keywords:** anesthesia, botulinum toxin, brachial plexus, chronic pain, ct-guided injection, neurogenic tos, pain management, pectoralis minor syndrome, thoracic outlet syndrome

## Abstract

Thoracic outlet syndrome is caused by compression of the neurovascular structures as they pass through the thoracic outlet and may present with pain, weakness, numbness, and paresthesias. Neurogenic thoracic outlet syndrome is the most common subtype and can be difficult to manage in patients with persistent symptoms despite conservative therapy and surgery. We present the case of a female patient in her 20s with post-traumatic neurogenic thoracic outlet syndrome following a motor vehicle accident, who continued to experience persistent pain and neurologic symptoms despite multiple surgical interventions, including first rib resection, scalenectomy, pectoralis minor tenotomy, and brachial plexus neurolysis. After recurrence of symptoms and failure of repeat surgical management, the patient underwent CT-guided botulinum toxin injection into the left pectoralis minor muscle following a successful diagnostic block. On follow-up, she reported approximately 60% improvement in pain, paresthesia, dysesthesia, grip strength, and daily functioning. Clinical improvement began approximately one week after the injection, was sustained for approximately six to seven months, and occurred without procedural complications. This case highlights the potential role of botulinum toxin injection as a minimally invasive therapeutic option in refractory neurogenic thoracic outlet syndrome, even after failed surgical treatment.

## Introduction

Thoracic outlet syndrome (TOS) refers to a collection of clinical findings and subjective symptoms caused by compression of the neurovascular bundle as it passes through the thoracic outlet. The thoracic outlet is the space between the axilla and the thorax that contains the brachial plexus, subclavian artery, and subclavian vein. These structures can be compressed in three main locations: the interscalene triangle above the clavicle, the costoclavicular space between the clavicle and first rib, and the coracopectoral tunnel (retropectoralis space) below the clavicle [1]. Anatomical variants such as anomalous cervical ribs, cervical fibrocartilaginous bands, supernumerary scalene muscles, and abnormal muscle insertions are common and can contribute to the development of TOS [2]. Acquired variations such as scalene, subclavius, or pectoralis minor muscle hypertrophy, chronic inflammatory change, repetitive injury, or trauma can also lead to TOS.

The term TOS encompasses three main types: neurogenic, venous, and arterial, with neurogenic TOS (nTOS) accounting for over 95% of cases [3]. Patients with nTOS endorse symptoms such as neck, arm, and hand pain, numbness, weakness, dysesthesia, and muscle atrophy caused by compression of the brachial plexus. Symptoms are often aggravated by activities requiring sustained use or elevation of the arm or hand. Provocative movements such as the Adson’s test, the elevated arm test, the upper limb tension test, and brachial plexus compression test can aid in diagnosis by replicating symptoms [1]. Additionally, chest and cervical spine radiography as well as computed tomography can help identify anatomic variance. Magnetic resonance neurogram is an emerging modality that can also be used to detect brachial plexus compression in nTOS [4].

Treatment is only indicated in symptomatic patients, as many patients with variable anatomy remain asymptomatic. Nonsurgical treatment is the first-line treatment modality, which includes physical therapy, pharmacologic agents, and injections (anesthetic, steroid, or botulinum toxin) [5-7].

Indications for thoracic outlet decompression surgery include progressive neurologic weakness, disabling pain or paresthesias, and failure of nonoperative management. However, considering the rate of recurrence and surgical complications, the benefit of surgical intervention remains questionable. Studies have reported recurrence rates of 15-20% after first rib resection and scalenectomy [6]. We report a case of a patient with post-traumatic nTOS with persistent pain following multiple surgeries and neurolysis who experienced significant pain relief following a left pectoralis minor botulinum toxin injection (BTI).

## Case presentation

A female patient in her 20s with left-sided post-traumatic nTOS that began after a whiplash-associated motor vehicle injury presented with left anterior shoulder and left chest wall pain.

The symptoms began in 2022 after a motor vehicle accident, in which she was hit by a speeding car as she was sitting in her stopped vehicle. After the injury, her symptoms included pain in her left shoulder, upper arm, and fingers. She also experienced weakness of her left hand, numbness, and tingling. She attempted physical therapy and pharmacologic agents including pregabalin, cyclobenzaprine, and lidocaine patches, but neither of these treatments gave her complete relief. After the failure of conservative management, she underwent a left first rib resection, neurolysis of the brachial plexus, scalenectomy, and pectoralis minor tenotomy in 2023. After her first surgery, she experienced meaningful relief of her symptoms for approximately two months before being rear-ended in a subsequent motor vehicle accident, which caused recurrence of her symptoms. A diagnostic left pectoralis minor muscle block with local anesthetic was performed and resulted in significant temporary improvement of her symptoms, supporting the pectoralis minor region as a pain generator. This was distinct from the later CT-guided BTI, which was performed as a therapeutic intervention.

She was diagnosed with recurrent left pectoralis minor syndrome and subsequently underwent re-operative resection of the left pectoralis minor muscle and repeat neurolysis of the left brachial plexus in 2024. However, her pain persisted despite surgical management. The patient underwent an MRI of the cervical spine, which revealed minimal degenerative changes without evidence of spinal canal or foraminal stenosis. Electromyography (EMG) was also performed, which was noted to be within normal limits, with no evidence of mononeuropathy, polyneuropathy, or radiculopathy. Importantly, there was no evidence of axonopathy affecting the lower trunk. Provocative maneuvers for TOS, including the elevated arm stress test, upper limb tension test, and brachial plexus compression test, were considered as part of the clinical evaluation. The patient’s overall presentation was consistent with recurrent nTOS in the setting of persistent left upper extremity pain, paresthesia, subjective weakness, prior surgical history, normal EMG findings, and symptomatic improvement after the diagnostic pectoralis minor muscle block.

Upon presentation to the interventional pain clinic, she reported persistent pain and subjective weakness. The physical examination was notable for left anterior shoulder pain and left upper chest wall pain that varied in intensity throughout the day. She also had allodynia and hyperalgesia in her left arm, left chest wall, palmar surface of her left hand, and fingers. She reported color changes in her left hand, with her hand turning purple after prolonged standing. She also reported that her left hand feels colder and intermittently spasms. Her pain was exacerbated by standing, movement, or lifting. She reported some relief with cyclobenzaprine, which was used as needed for muscle spasms, and lidocaine patches, which helped with sensitivity. The patient was previously also taking pregabalin; however, it was discontinued due to reported brain fog. She rated her average daily pain as a 2-3 on a 10-point Numeric Rating Scale (NRS), where 0 represents no pain and 10 represents the worst pain imaginable [8]. However, she reported intermittent flares to 4-5/10. She also described her symptoms as similar to those experienced before her first procedure, though not as severe. CT-guided left pectoralis minor BTI was performed under image guidance (Figure 1).

**Figure 1:**
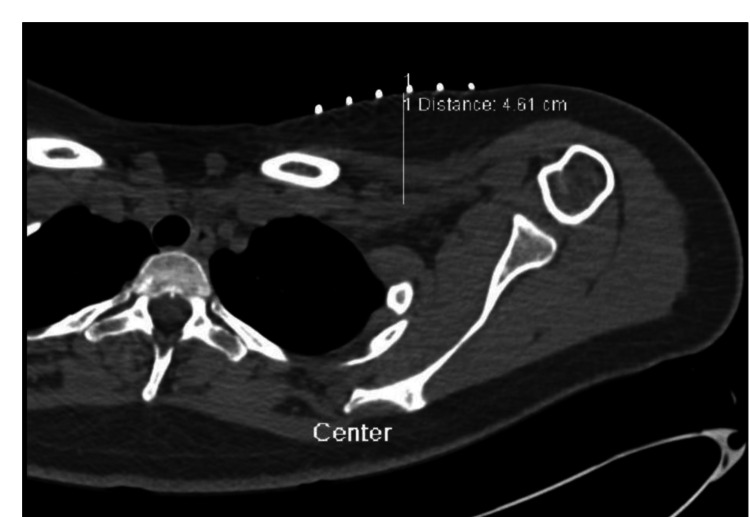
CT-guided left pectoralis minor botulinum toxin injection CT image demonstrating image-guided needle placement and trajectory for botulinum toxin injection targeting the left pectoralis minor region in a patient with refractory neurogenic thoracic outlet syndrome. CT, computed tomography

At the follow-up, the patient reported approximately 60% improvement in her symptoms following the CT-guided BTI. Improvement began approximately one week after the procedure and included decreased pain, paresthesia, dysesthesia, numbness, subjective weakness, and intermittent muscle spasms, with associated improvement in grip strength. She also reported improved ability to perform activities of daily living, including grooming, bathing, and other routine tasks involving the upper extremities. The clinical benefit was sustained for approximately six to seven months based on subsequent follow-ups. As symptoms gradually recurred, she was scheduled for a repeat BTI. No procedural complications or treatment-related adverse effects were reported.

## Discussion

Treatment of symptomatic TOS often begins with conservative management including patient education, NSAIDs, and physical therapy. Physical therapy focuses on range-of-motion exercises, as well as improving body mechanics and ergonomics to decrease pressure on the neurovascular structures [7]. If physical therapy and medication management do not resolve symptoms, BTIs can be considered. The muscles that are most frequently injected are the anterior scalene, middle scalene, and pectoralis minor. In cases of refractory nTOS, surgery can be considered, though widely accepted as a last resort.

All surgical procedures for nTOS aim to relieve the compression on the brachial plexus and thereby alleviate the symptoms. Several studies have evaluated BTI techniques and outcomes in nTOS [9-11]. There are multiple surgical options, but a first rib resection with scalenectomy is most established in the literature [12,13]. This surgery involves removing compressive structures (the first rib, scalene muscles, and fibrous bands) from the thoracic outlet, directly decompressing the brachial plexus and allowing for complete neurolysis. Rib-sparing scalenectomy is a surgery that involves resection of the anterior and middle scalene muscles and neurolysis without removing the first rib [14]. Isolated pectoralis minor tenotomy can also be used for patients with compression that is localized to the retropectoralis muscle space [15]. This procedure decompresses the brachial plexus as it passes underneath the muscle by releasing the pectoralis muscle tendon [15]. Surgical outcomes with all of these approaches vary widely. Studies show recurrence of symptoms rates ranging from 15% to 30% post-surgery [16-19]. Initial surgical success rates have been shown to be between 91% and 93%, but at 10 years post-surgery, success rates range from 64% to 71% [17]. Some studies have made the argument that surgery does not improve long-term functional outcomes when compared to conservative treatment [18]. Trigger point injections with local anesthetic into the trapezius muscle are an alternate pharmacologic strategy. The reasons for these surgical failures are not clear but could be secondary to difficulty identifying the exact etiology of the patient’s pain or the development of new pain generators post-surgery.

In this case, our patient failed surgical management and experienced recurrent symptoms after left first rib resection, neurolysis of the brachial plexus, scalenectomy, and pectoralis minor tenotomy. However, following CT-guided BTI, she experienced approximately 60% improvement in pain and neurologic symptoms, with associated improvement in daily functioning. Clinical benefit began approximately one week after treatment, persisted for six to seven months, and was achieved without procedural complications. As symptoms gradually recurred, she was scheduled for a repeat BTI, suggesting that BTI may provide a safe and effective minimally invasive treatment option for patients with refractory nTOS after failed surgical management. Onabotulinum toxin A works by inhibiting presynaptic acetylcholine release at the neuromuscular junction, resulting in chemodenervation [11]. In cases of nTOS, this muscle paralysis and reduction in muscle tone decrease the compression of the neurovascular bundle and relieve symptoms [5,11]. The anterior scalene muscle is the most commonly injected as it forms the anterior border of the interscalene triangle where the brachial plexus is often compressed. The middle scalene muscle is less frequently injected, but it can be combined with the anterior scalene muscle in complicated cases of nTOS. The middle scalene muscle forms the posterior border of the interscalene triangle [1]. If the brachial plexus compression is located in the retropectoralis space, then injecting the pectoralis minor muscle relieves tension in the muscle and decompresses the brachial plexus. Additionally, adjunct sites can also be injected. Studies have shown a reduction of symptoms after BTI in 46-84% of patients [9-12]. The duration of symptom relief varies from several weeks up to six months [9,11].

The current literature extensively examines BTI presurgery, with some studies showing that a better response to BTI positively correlates with better responses to surgery [20]. The subclavius, trapezius, and levator scapulae muscles have been described in the literature with varying responses [21]. The amount of onabotulinum toxin A that is used in these locations ranges widely from 10 to 100 units [21]. This may reflect the ability of botulinum toxin injections to help identify the specific muscle responsible for neurovascular compression. If a patient experiences symptomatic improvement after injection of a particular muscle, surgical treatment targeting that same muscle may be more likely to provide lasting symptom relief. A targeted literature search was performed using combinations of terms: “neurogenic thoracic outlet syndrome”, “botulinum toxin injection”, “failed surgical decompression”, “recurrent thoracic outlet syndrome”, “first rib resection”, “scalenectomy”, and “pectoralis minor tenotomy”. Although prior studies and systematic reviews have described BTI as a diagnostic or therapeutic tool in nTOS, we did not identify a published case specifically describing CT-guided pectoralis minor BTI after failed surgical decompression, including prior first rib resection, scalenectomy, pectoralis minor tenotomy, and brachial plexus neurolysis. Therefore, this case appears to represent a rarely reported post-surgical application of BTI in refractory nTOS.

Our patient's case highlights a rarely reported post-surgical use of BTI and emphasizes the importance of considering BTI as part of the treatment algorithm for refractory nTOS after failed surgery. The established treatment algorithm for nTOS typically begins with conservative and medication management using NSAIDs, trigger point injections, behavior modification, and physical therapy. BTI may then be considered, and patients with persistent symptoms are often referred for surgical care. In this case, however, the patient had persistent symptoms despite multiple surgical interventions, while CT-guided pectoralis minor BTI was associated with approximately six to seven months of symptomatic and functional improvement. Although this case is limited to one patient, the clinical course suggests that repeat BTI may be considered when symptoms recur. Longer-term follow-up is still needed to determine the durability of repeated injections and whether additional muscles in the thoracic outlet may need to be targeted in the future. This case is also limited by the absence of standardized objective post-treatment outcome measures, detailed post-injection strength testing, and formal adverse event assessment. Available follow-up was based on patient-reported symptomatic improvement, and future studies should include validated functional outcome measures, standardized pain scores, objective strength assessments, adverse event monitoring, and longer-term follow-up to better define the durability and safety of BTI in refractory nTOS.

## Conclusions

This case report highlights the potential role of BTI in the treatment of refractory nTOS following failed surgical management. In this patient, treatment was associated with sustained symptom relief, improved daily functioning, and no procedural complications, supporting BTI as a minimally invasive therapeutic option in carefully selected patients. Further studies with standardized outcome measures and longer-term follow-up are needed to better define its durability, safety, and role in the treatment algorithm for refractory nTOS.
